# H^+^‐pyrophosphatase IbVP1 promotes efficient iron use in sweet potato [*Ipomoea batatas* (L.) Lam.]

**DOI:** 10.1111/pbi.12667

**Published:** 2017-02-10

**Authors:** Weijuan Fan, Hongxia Wang, Yinliang Wu, Nan Yang, Jun Yang, Peng Zhang

**Affiliations:** ^1^ National Key Laboratory of Plant Molecular Genetics CAS Center for Excellence in Molecular Plant Sciences Institute of Plant Physiology and Ecology Shanghai Institutes for Biological Sciences Chinese Academy of Sciences Shanghai China; ^2^ Shanghai Key Laboratory of Plant Functional Genomics and Resources Shanghai Chenshan Plant Science Research Center Chinese Academy of Sciences Shanghai Chenshan Botanical Garden Shanghai China

**Keywords:** sweet potato, iron acquisition, H^+^‐pyrophosphatase, rhizosphere acidification, auxin regulation, carbohydrate metabolism

## Abstract

Iron (Fe) deficiency is one of the most common micronutrient deficiencies limiting crop production globally, especially in arid regions because of decreased availability of iron in alkaline soils. Sweet potato [*Ipomoea batatas* (L.) Lam.] grows well in arid regions and is tolerant to Fe deficiency. Here, we report that the transcription of type I H^+^‐pyrophosphatase (H^+^‐PPase) gene *IbVP1* in sweet potato plants was strongly induced by Fe deficiency and auxin in hydroponics, improving Fe acquisition via increased rhizosphere acidification and auxin regulation. When overexpressed, transgenic plants show higher pyrophosphate hydrolysis and plasma membrane H^+^‐ATPase activity compared with the wild type, leading to increased rhizosphere acidification. The *IbVP1*‐overexpressing plants showed better growth, including enlarged root systems, under Fe‐sufficient or Fe‐deficient conditions. Increased ferric precipitation and ferric chelate reductase activity in the roots of transgenic lines indicate improved iron uptake, which is also confirmed by increased Fe content and up‐regulation of Fe uptake genes, e.g. *FRO2*, *IRT1* and *FIT*. Carbohydrate metabolism is significantly affected in the transgenic lines, showing increased sugar and starch content associated with the increased expression of *AGPase* and *SUT1* genes and the decrease in β‐amylase gene expression. Improved antioxidant capacities were also detected in the transgenic plants, which showed reduced H_2_O_2_ accumulation associated with up‐regulated ROS‐scavenging activity. Therefore, H^+^‐PPase plays a key role in the response to Fe deficiency by sweet potato and effectively improves the Fe acquisition by overexpressing *IbVP1* in crops cultivated in micronutrient‐deficient soils.

## Introduction

Iron (Fe) deficiency inhibits plant growth and reduces crop yields and quality, especially in alkaline and calcareous soils. Approximately 30% of the world's cultivated soil is susceptible to Fe deficiency due to severely limited bioavailability of Fe (Guerinot and Yi, 1994). In plants grown under Fe deficiency, the ferric chelate reductase (FCR) and H^+^‐ATPase (PM‐ATPase) activity are up‐regulated in the plasma membrane. In addition, the expression of other Fe uptake genes is increased in response to altered levels of hormones (e.g. auxin; Schmidt *et al*., 2000; Santi and Schmidt, 2008, 2009; Bacaicoa *et al*., 2011; Kabir *et al*., 2013), carbohydrates (e.g. sucrose; Zargar *et al*., 2015; Lin *et al*., 2016) and reactive oxygen species (ROS; Molassiotis *et al*., 2006; Donnini *et al*., 2011), explaining the diverse effects of Fe deficiency on plant development and growth (Briat *et al*., 2015; Kobayashi and Nishizawa, 2012).

Sweet potato [*Ipomoea batatas* (L.) Lam.] is an important root crop ranking seventh in the annual production worldwide. It plays a key role in food security and nutritional intervention, especially in Africa and Asia (Bovell‐Benjamin, 2007; Low, 2011). Sweet potato thrives in both fertile‐ and nutrient‐deficient soils (Bovell‐Benjamin, 2007; Woolfe, 1992). Most lands used for its cultivation are relatively infertile and lack bioavailable Fe (White and Zasoski, 1999; Yan *et al*., 2006; Zuo and Zhang, 2011). Therefore, genetic improvement in sweet potato is essential to enhance tolerance to iron deficiency and increase iron content in storage roots (White and Broadley, 2009). Plants absorb iron from the soil via increased plasmalemma FCR activity in the roots. Fe absorption is also enhanced by PM‐ATPase‐mediated proton extrusion that acidifies the root apoplast and rhizosphere, as well as activation of high‐affinity transport systems (Grusak and Pezeshgi, 1996; Jin *et al*., 2007, 2008, 2009). Unfortunately, no such study was conducted in sweet potato. Adamski *et al*. (2011, 2012) recently reported the effect of different iron concentrations on the morphological, anatomical, enzymatic, physiological and photosynthetic characteristics of sweet potato. It is necessary to study the response of sweet potato to Fe deficiency and develop useful approaches to increase Fe acquisition from the soil.

The H^+^‐translocating inorganic pyrophosphatase (H^+^‐PPase) plays a key role in plant energy metabolism by hydrolysing pyrophosphate (PPi) into sodium and/or proton gradients and transporting these ions across the membrane (Luoto *et al*., 2013). The hydrolytic activity balances the cytoplasmic pyrophosphate generated in various anabolic reactions such as DNA, RNA and protein synthesis in which PPi released as a by‐product of ATP hydrolysis, and inhibits gluconeogenesis and cellulose synthesis (Baykov *et al*., 1999). Up‐regulation of H^+^‐PPase expression and activity has been reported in plants grown under abiotic stress conditions such as chilling, anoxia, mineral deficiency and salt stress (Maeshima, 2000). Up‐regulated H^+^‐PPases induce plant tolerance to various stresses, such as drought, high salinity, and N and Pi deprivation (Arif *et al*., 2013; Paez‐Valencia *et al*., 2013; Yang *et al*., 2007). It also affects auxin and sugar transport and accumulation during root and shoot growth (Gaxiola *et al*., 2012; Khadilkar *et al*., 2016; Li *et al*., 2005; Pasapula *et al*., 2011; Pizzio *et al*., 2015).

The H^+^‐PPase overexpression enhances apoplastic acidification by increasing the abundance and activity of H^+^‐ATPase at the plasma membrane (Gaxiola *et al*., 2016; Li *et al*., 2005; Undurraga *et al*., 2012). It increases root and shoot biomass, photosynthetic capacity and nutrient uptake under normal or nutrient‐limited conditions, such as low NO^3−^ and Pi (Khadilkar *et al*., 2016; Li *et al*., 2005; Lv *et al*., 2015; Paez‐Valencia *et al*., 2013; Pizzio *et al*., 2015; Yang *et al*., 2007, 2014). Rhizosphere acidification is a principal mechanism in plant mineral nutrition, because it contributes to nutrient solubility and proton‐motive force in the plasma membrane (Palmgren, 2001). Acidification also accelerates auxin transport to increase lateral root branching via up‐regulated H^+^‐ATPase and FCR activity (Cho and Hong, 1995; Frías *et al*., 1996; Li *et al*., 2000; Zheng *et al*., 2003), and secretion of organic acids (Ruan *et al*., 2013; Yang *et al*., 2007), suggesting increased resilience to Fe deficiency. Nevertheless, the role of H^+^‐PPase in enhancing Fe absorption in plants is largely unknown.

In this study, we reported the functional characterization of a single‐copy sweet potato H^+^‐PPase gene *IbVP1* to improve Fe uptake and utilization via increased rhizosphere acidification. Overexpression of *IbVP1* in sweet potato increases auxin transport, sugar content and ROS scavenging reflecting the intrinsic mechanisms of Fe acquisition in this important hexaploid species.

## Results

### *IbVP1* is single‐copied type I H^+^‐PPase gene in hexaploid sweet potato

**Figure 1:**
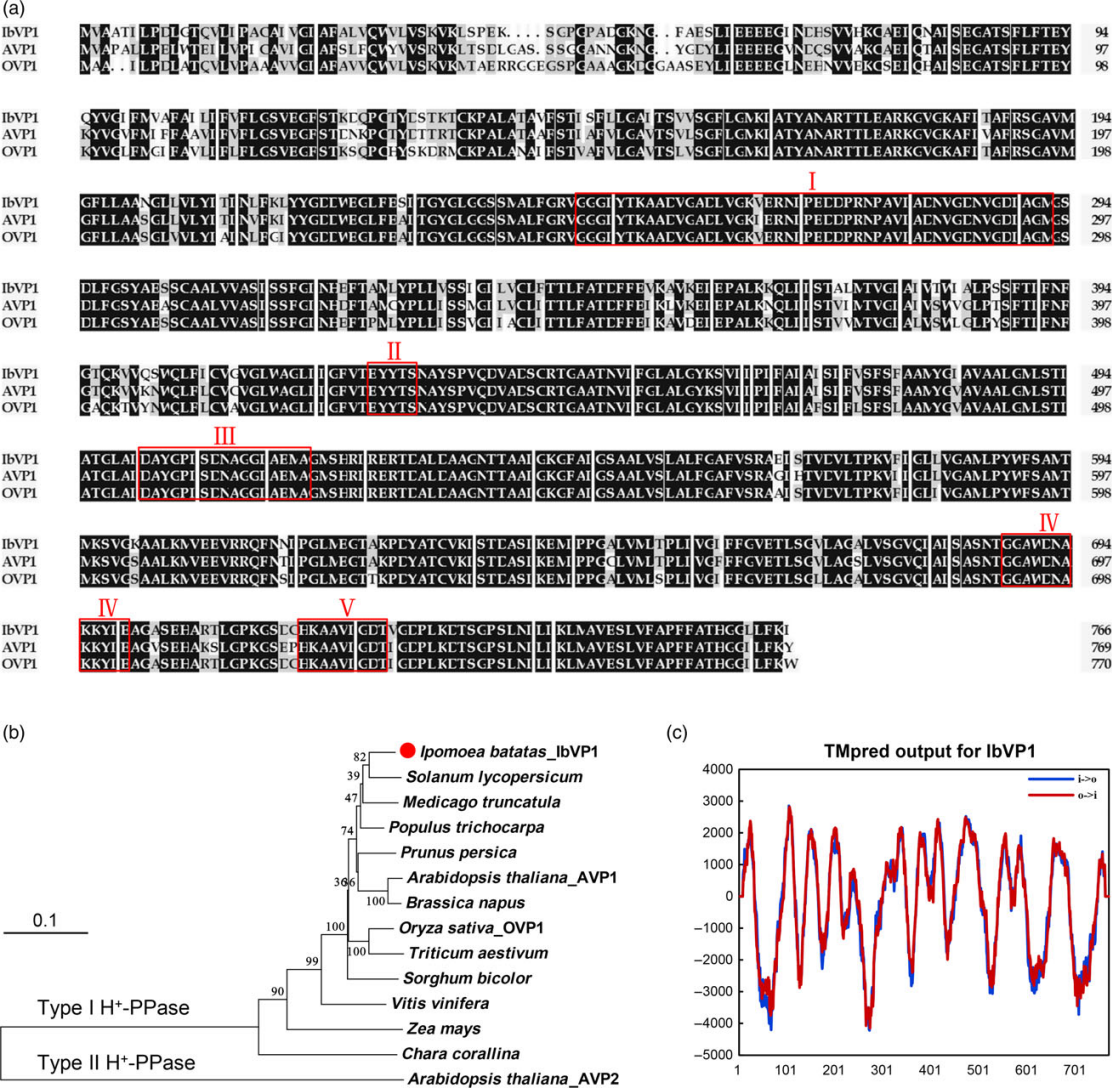
Amino acid sequence alignment and phylogenetic analysis of IbVP1 compared with other species. (a) Multiple alignment of the deduced amino acid sequences of H^+^‐PPase proteins from *Ipomoea batatas* (IbVP1, AFQ00710.1), *Arabidopsis thaliana* (AVP1, NP_173021.1) and *Oryza sativa* (OVP1, BAA08232.1). Residues are highlighted in black and grey according to the level of conservation. The highly conserved motifs reported by Drozdowicz and Rea (2001) for H^+^‐PPase proteins are boxed. (b) Phylogenetic tree of typical vacuolar H^+^‐PPase proteins derived from various species, including *Medicago truncatula* (XP_003609463), *Brassica napus* (NP_001302829.1), *Populus trichocarpa* (XP_006381091.1), *Prunus persica* (AF367446_1), *Solanum lycopersicum* (BAM65603.1), *Sorghum bicolor* (BAM65603), *Triticum aestivum* (ABX10014.1), *Vitis vinifera* (NP_001268072.1), *Zea mays* (NP_001152459.1) and *Arabidopsis thaliana* (AVP2, ABX10014.1). (c) The predicted transmembrane domains of IbVP1 protein using TMpred program.

The full‐length cDNA of *IbVP1* gene consists of 2644 nucleotides and encodes a predicted polypeptide of 737 amino acids with a molecular weight of 77.7 kDa and pI 5.84. Amino acid sequence alignment reveals that the IbVP1 shares high level of identity with H^+^‐PPases in other plant species, such as the dicot *Arabidopsis thaliana* (85.5%) and the monocot *Oryza sativa* (85.3%), which contain the highly conserved domains reported by Drozdowicz and Rea (2001) (Figure 1a). Further, phylogenetic analysis suggests that IbVP1 belongs to vacuolar H^+^‐pyrophosphatases and is clustered to type I (K^+^‐sensitive) H^+^‐PPases (Figure 1b). IbVP1 was shown to contain 13 conserved membrane‐spanning domains using TMpred program for transmembrane prediction (Figure 1c). Southern blot of genomic DNA digested with *Eco*RV, *Xba*I, *Xho*I and *Hin*dIII revealed a single band when probed with the 5′‐UTR of *IbVP1*, indicating that *IbVP1* is a single‐copied gene in the hexaploid species (Figure S1).

### *IbVP1* is ubiquitously expressed and induced by Fe deficiency and auxin in sweet potato

**Figure 2:**
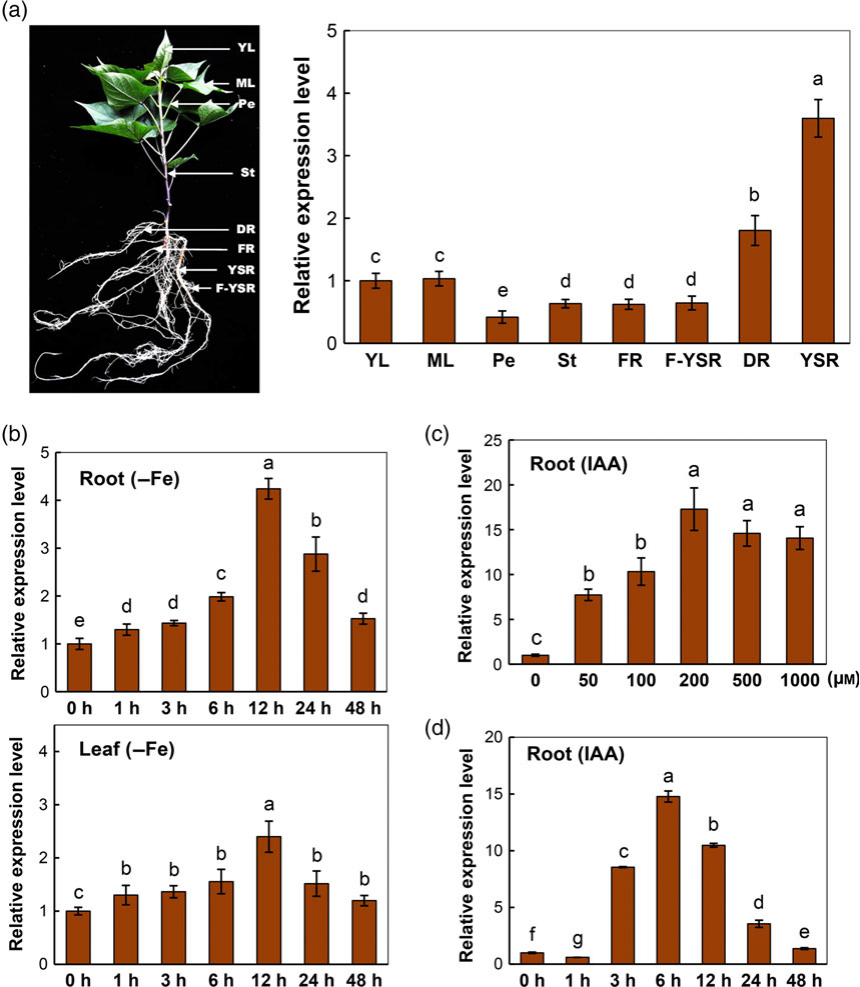
The *IbVP1* expression in sweet potato and in response to Fe deficiency (–Fe) and auxin treatment. (a) Expression pattern of *IbVP1* by qRT‐PCR analyses using sweet potato tissues of plants at 50 d after planting; YL, young leaf; ML, mature leaf; Pe, petiole; St, stem; FR, fibrous root (diameter <0.2 cm); F‐YSR, fibrous root from young storage root; DR, developing root (0.2 cm < diameter < 0.5 cm); YSR, young storage root (diameter 0.5–1.0 cm). (b) Time‐course response of *IbVP1* expression in sweet potato under Fe condition in fibrous roots and leaves at various time periods (0–48 h). (c) Transcriptional variation in *IbVP1* in response to treatment with various concentrations of exogenous IAA for 3 h in sweet potato roots. (d) *IbVP1* expression in response to IAA (200 μm) for various time periods (0–48 h) in sweet potato roots. qRT‐PCR data were normalized to those for the endogenous *Actin* gene. Error bars indicate the standard deviation between three technical replicates measured in fibrous roots and leaves collected from at least three different plantlets and subsequently pooled for analysis. Different letters indicate significant differences (one‐way ANOVA,* P *< 0.05).

To determine the expression pattern in various tissues of greenhouse‐grown sweet potato plant, qRT‐PCR and semi‐quantitative RT‐PCR analyses were performed in the leaf, petiole, stem, fibrous root and storage root tissues at three different developmental stages: fibrous root (diameter <0.2 cm), developmental root (0.2 cm < diameter <0.5 cm) and young storage root (diameter 0.5–1.0 cm), denoted by FR, DR and YSR (Figure 2a). *IbVP1* transcription was detected in all tissues, with the highest expression in young storage root tissues (Figures 2a and S2a), indicating its role in storage root development.

In a 48‐h Fe deficiency regimen using hydroponic growth system, the transcripts of *IbVP1* in both roots and leaves were significantly induced by Fe deficiency, reaching a maximum at 12 h followed by a gradual decrease (Figures 2b and S2b). A fourfold increase in *IbVP1* transcription in root was observed at 12 h, and twofold higher in leaf at 12 h, when compared with that at 0 h. Exogenous auxin treatment of sweet potato in a solution containing various concentrations of 3‐indole acetic acid (IAA; 0, 50, 100, 200, 500 and 1000 μm) for 3 h also dramatically up‐regulated the *IbVP1* transcription (Figures 2c and S2c). A maximum transcript level (~17‐fold that of control) was detected with 200 μm IAA (Figure 2c). A time‐course study of *IbVP1* expression (0, 1, 3, 6, 12, 24 and 48 h) in fibrous roots cultured in 200 μm IAA solution revealed that the maximum *IbVP1* transcription level was detected at 6 h after treatment (Figures 2d and S2d), which was about 15‐fold higher than the initial levels. These results suggest that the transcription of *IbVP1* is extensively regulated in response to exogenous IAA. Taken together, the strong *IbVP1* response in Fe deficiency and auxin suggests its critical role in plant response to Fe deficiency and plant growth.

### *IbVP1*‐overexpressed sweet potato shows enhanced V‐PPase and PM‐ATPase activities

At least nine independent transgenic lines of Taizhong 6 cultivar were generated using the *Agrobacterium*‐mediated transformation and confirmed by Southern blot for T‐DNA integration (Figure S3a,b) and by qRT‐PCR for *IbVP1* expression (Figure S3c). Three single‐integrated transgenic lines IA4, IA7 and IA8 with the highest levels of expression showed normal phenotype and growth and were used in the following study.

**Figure 3:**
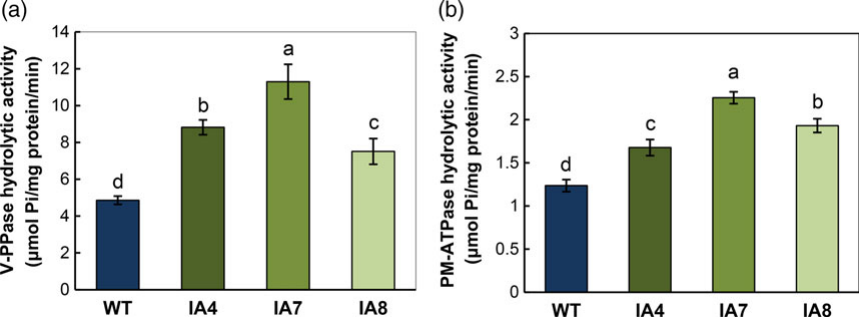
Hydrolytic activities of vacuolar H^+^‐PPase and PM‐H^+^‐ATPase in sweet potato plants. (a) Vacuolar H^+^‐PPase activities of root tonoplast vesicles monitored by the release of Pi from PPi; (b) PM‐H^+^‐ATPase activities in root microsomal fractions monitored by phosphate release. IA,* IbVP1* transgenic line; WT, wild‐type plant. Data represent means ± SD of three independent assays. Different letters indicate significant differences (one‐way ANOVA,* P *<* *0.05).

Consistent with *IbVP1* expression, the three transgenic plants overexpressing *IbVP1* also showed higher V‐H^+^‐PPase activities in the tonoplasts of the root apex than in the WT under normal conditions (Figure 3a). Further, the PM‐H^+^‐ATPase hydrolytic activities in root microsomal fractions were also increased, at least 35% higher, in IA transgenic plants than in WT (Figure 3b), indicating the coupling effect between H^+^‐PPase and PM‐H^+^‐ATPase activities (Undurraga *et al*., 2012).

### Overexpression of *IbVP1* promotes root development

**Figure 4:**
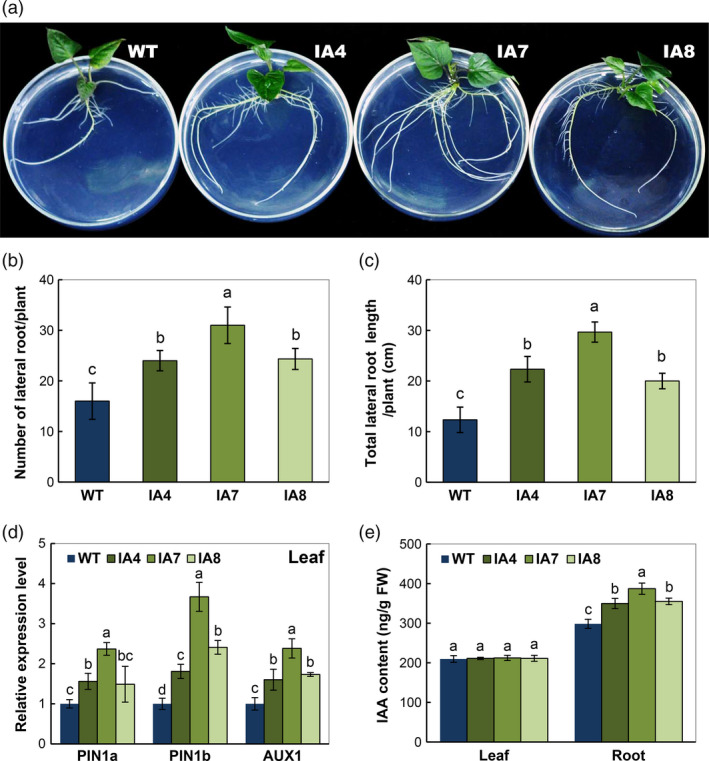
Growth status and auxin response in sweet potato plants. (a) Phenotypes in normal MS solid medium; (b, c) lateral root characters; (d) expression of auxin transport‐associated genes in leaves; (e) indole‐3‐acetic acid (IAA) content of roots and leaves. IA,* IbVP1* transgenic line; WT, wild‐type plant. Values represent means ± SD using three plants per line of three independent experiments. Different letters indicate significant differences (one‐way ANOVA,* P *<* *0.05).

Under normal conditions, the transgenic plants exhibited improved plant morphology compared with the WT plants (Figure 4a), noticeably in root. The IA transgenic plants produced more lateral roots (65% in average, Figure 4b) and longer lateral root length (95% in average, Figure 4c) compared with WT. To validate the increased root system following auxin flux in the transgenic plants, the expression of several key genes involved in auxin polar transport was analysed by qRT‐PCR. Higher expression of *PIN1a* and *PIN1b*, the two genes encoding auxin efflux carriers, and *AUX1,* encoding an auxin influx carrier, were detected in IA transgenic plants compared with WT (Figure 4d). Accordingly, IAA content was significantly increased in the roots of IA transgenic plants (Figure 4e). No significant difference in leaf IAA content was found between IA plants and WT, indicating that the enhanced root growth was due to increased auxin transport from shoot into root in transgenic plants, which is consistent with the previous observations of *AVP1*‐overexpressing Arabidopsis plants (Li *et al*., 2005).

### Overexpression of *IbVP1* increases tolerance of transgenic plants to Fe deficiency

**Figure 5:**
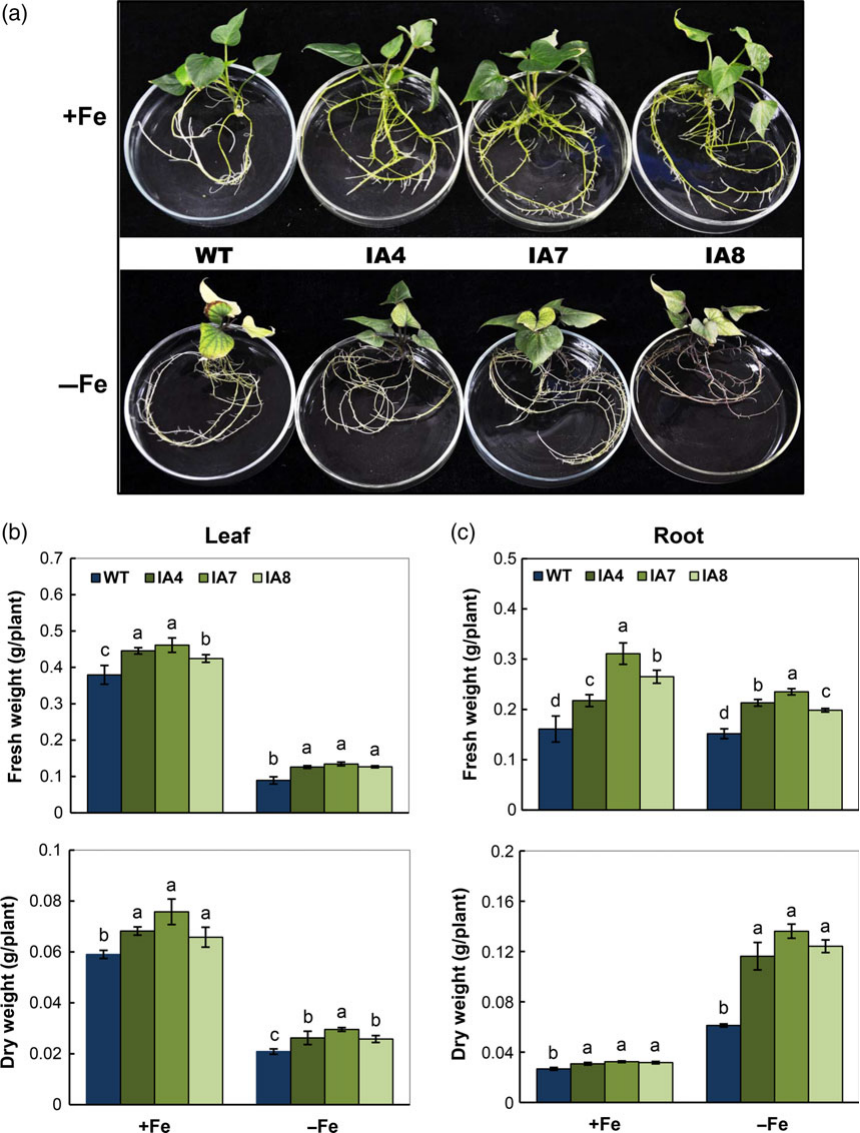
Growth response to Fe deficiency in sweet potato plants cultured *in vitro*. (a) Growth phenotype under exposure to Fe‐sufficient (+Fe) or Fe‐deficient (−Fe) medium after 15 days of culture; (b, c) fresh weights and dry weights of leaves (b) and roots (c). IA,* IbVP1* transgenic line; WT, wild‐type plant. Values represent means ± SD using three plants per line of three independent experiments. Different letters indicate significant differences (one‐way ANOVA,* P *<* *0.05).

Under Fe deficiency, the IA lines showed better plant growth in solid medium with more greenish leaves and lateral roots, unlike the WT with obvious chlorotic leaves and weak roots. Even under Fe sufficiency, the differences in plant phenotype were obvious, with more lateral roots in the IA plants (Figure 5a). Increase in the leaf and root biomass was detected in IA plants under both conditions (Figure 5b,c) and more significantly in roots under the Fe deficiency condition. Under adequate Fe levels, the leaf biomass increase in transgenic lines ranged from 10.5% to 21.1% in fresh weight (FW) and from 11.9% to 28.8% in dry weight (DW) compared with that of WT. In roots, the biomass increase in transgenic lines ranged from 35.4% to 93.2% in FW and from 14.8% to 18.5% in DW. Under Fe deficiency, the leaf biomass increase in transgenic lines ranged from 41.6% to 50.6% in FW and from 23.8% to 42.9% in DW compared with that of WT. In roots, the biomass increase in transgenic lines ranged from 30.3% to 54.6% in FW and from 90.2% to 123% in DW (Figure 5b,c).

**Figure 6:**
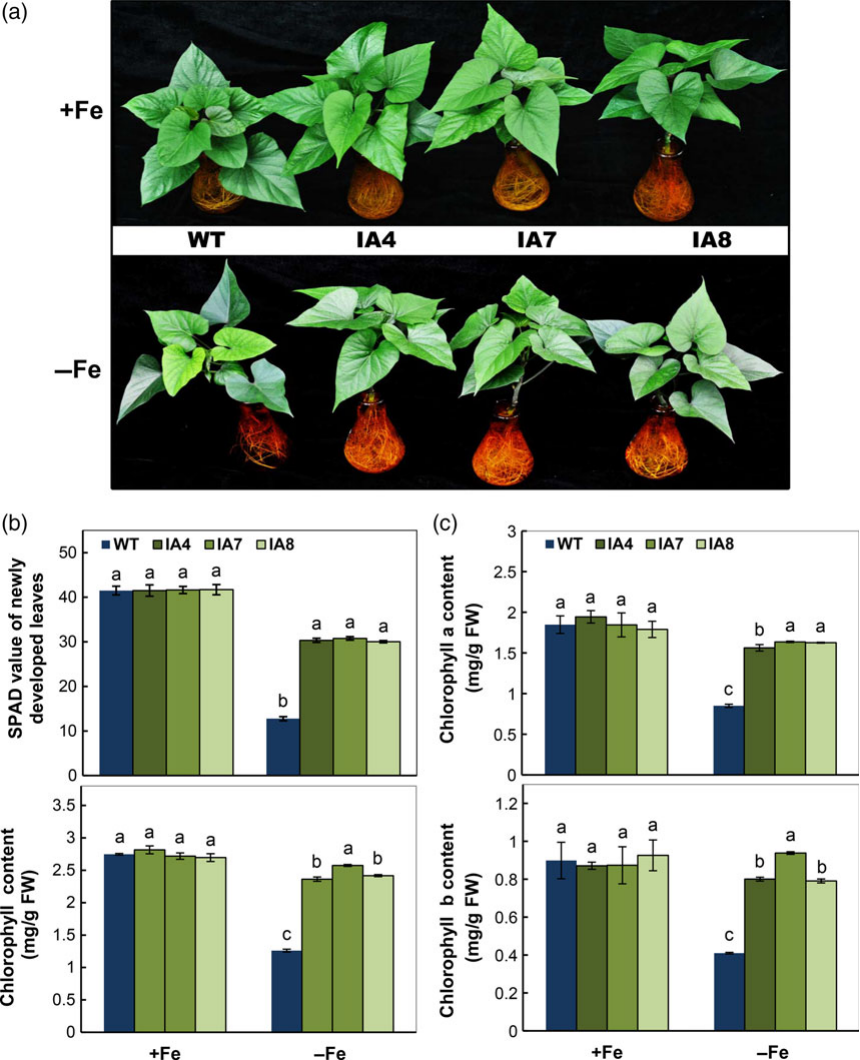
Growth and photosynthetic response to Fe deficiency in sweet potato plants in hydroponic culture. (a) Growth status under Fe‐sufficient (+Fe) or Fe‐deficient (−Fe) conditions; (b) SPAD index and total chlorophyll content of the newly developed leaves; (c) contents of chlorophyll a and chlorophyll b. IA,* IbVP1* transgenic line; WT, wild‐type plant. Values represent means ± SD using three plants per line of three independent experiments. Different letters indicate significant differences (one‐way ANOVA,* P *<* *0.05).

When the IA transgenic and WT plants were grown in hydroponic Fe sufficiency condition for 2 weeks and transferred to Fe‐sufficient or Fe‐deficient hydroponic solution for another 2 weeks, leaf chlorosis was observed in newly emerging WT leaves (Figure 6a). Approximately 2.3‐fold‐reduced SPAD reading was found in chlorotic leaves. The transgenic plants were only reduced about 37% compared with plants grown under Fe‐sufficient conditions (Figure 6b), which is also reflected by the significant decrease in total chlorophyll content (Figure 6b, bottom panel), including chlorophyll a and b (Figure 6c).

### Overexpression of *IbVP1* promotes rhizosphere acidification and Fe acquisition

**Figure 7:**
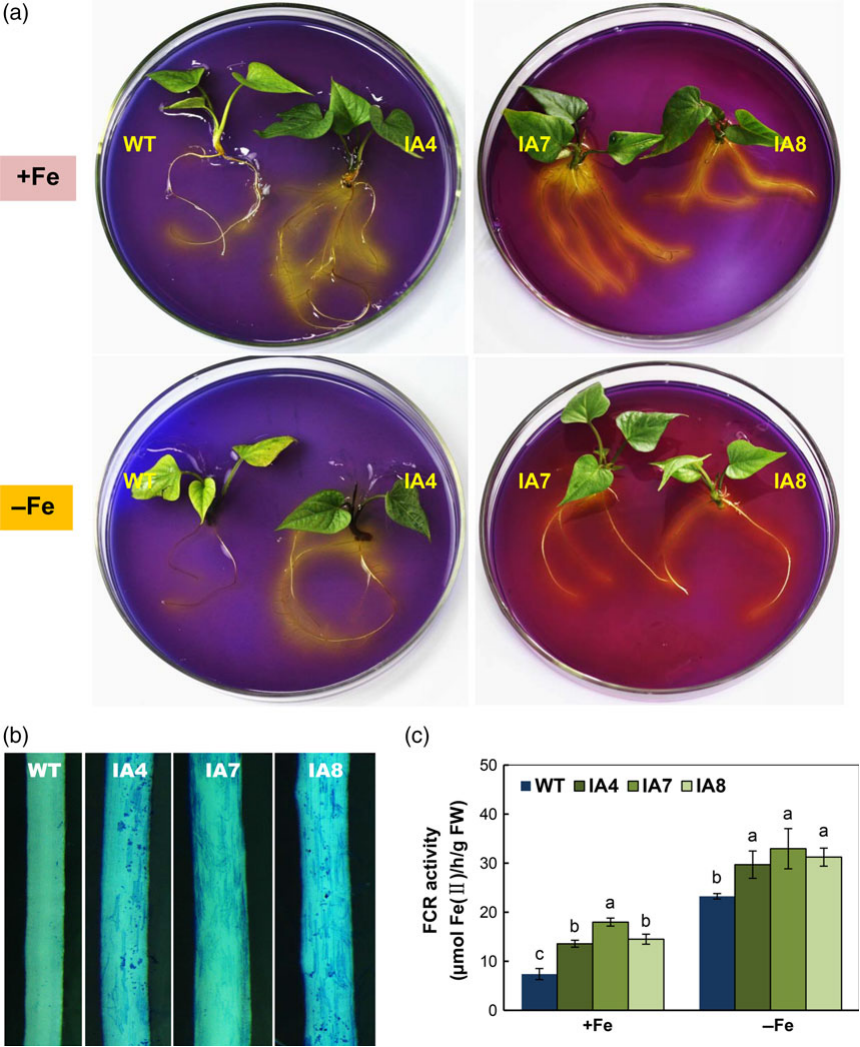
Fe acquisition analysis of sweet potato plants in response to Fe deficiency. (a) Rhizosphere acidification assay using agar plates containing bromocresol purple for 4 h; (b) root ferric precipitation assay of IA plants grown on Fe‐sufficient (+Fe) medium with Perls’ Prussian blue staining; (c) ferric chelate reductase (FCR) activity in the roots of plants grown under Fe‐sufficient (+Fe) or Fe‐deficient (−Fe) conditions. IA,* IbVP1* transgenic line; WT, wild‐type plant. Values represent means ± SD using three plants per line of three independent experiments. Different letters indicate significant differences (one‐way ANOVA,* P *<* *0.05).

As indicated above, IA transgenic lines show enhanced V‐PPase and PM‐ATPase hydrolytic activities (Figure 3). Theoretically, rhizosphere acidification is increased in IA lines. Indeed, compared with WT, IA plants showed greater acidification zone indicated by the yellow colour in agar plates containing the pH indicator bromocresol purple for 4 h, regardless of iron levels (Figure 7a). Further, Perls’ Prussian blue staining analysis of root Fe^3+^ precipitation also confirmed increased Fe^3+^ intensity in the IA plants (Figure 7b). The root FCR activity was increased in the IA lines under both Fe‐sufficient and Fe‐deficient conditions (Figure 7c).

**Figure 8:**
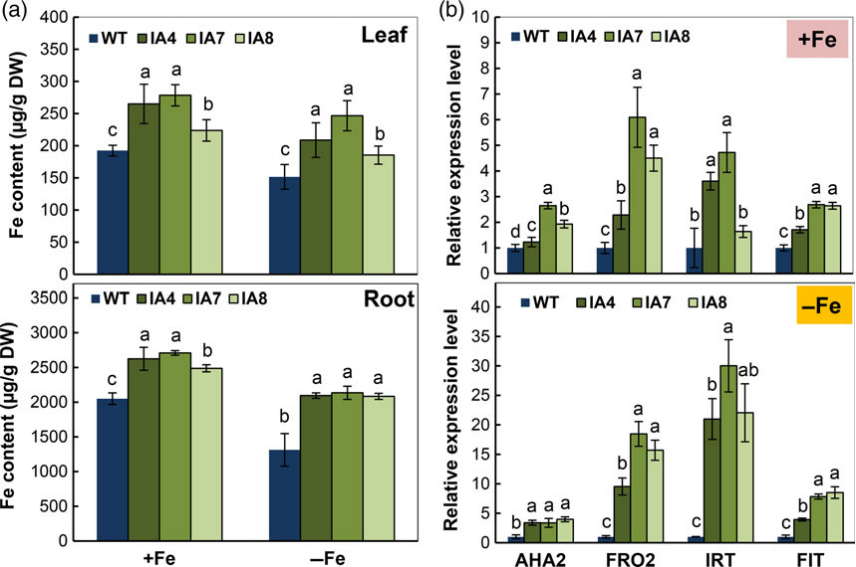
Iron accumulation and expression profile of Fe uptake genes in sweet potato grown under Fe‐sufficient (+Fe) and Fe‐deficient (−Fe) conditions. (a) Fe content in leaves and roots; values represent means ± SD using three plants per line in three independent experiments. Different letters indicate significant differences (one‐way ANOVA,* P *<* *0.05); (b) qRT‐PCR analysis of altered expression of H^+^‐ATPase gene *AHA2*, FERRIC REDUCTION OXIDASE2 (*FRO2*), IRON‐REGULATED TRANSPORTER1 (*IRT1*) and FER‐LIKE FE DEFICIENCY‐INDUCED TRANSCRIPTION FACTOR (*FIT*). IA,* IbVP1* transgenic line; WT, wild‐type plant. Values were normalized to those of the endogenous *Actin* gene. Error bars indicate standard deviation between three technical replicates measured in fibrous roots collected from at least three different sweet potato plantlets and subsequently pooled for analysis.

Fe contents in the young leaves and roots of IA lines were significantly higher than in the WT (Figure 8a), under Fe‐sufficient or Fe‐deficient conditions. The average increase in Fe content of IA lines was 40.9% in leaves and 60.3% in roots compared with that of WT under Fe‐deficient conditions. Notably, a ten‐fold difference in Fe content was found between leaf and root. The highest Fe content was detected in the roots of IA7, reaching 2709 and 2135 μg/g (DW) under Fe sufficiency or Fe deficiency, respectively. Further, the expression of Fe acquisition genes that are involved in Fe uptake and proton release was also affected. The qRT‐PCR analysis of *FRO2*,* IRT1*,* FIT* and H^+^‐ATPase gene *AHA2* revealed a dramatic increase in the expression of IA transgenic plants, grown under Fe deficiency (Figure 8b). All these findings suggested up‐regulation of Fe uptake in the IA plants. These results indicate that *IbVP1* promoted Fe acquisition in sweet potato.

### *IbVP1*‐overexpression enhances antioxidant activity under Fe deficiency

**Figure 9:**
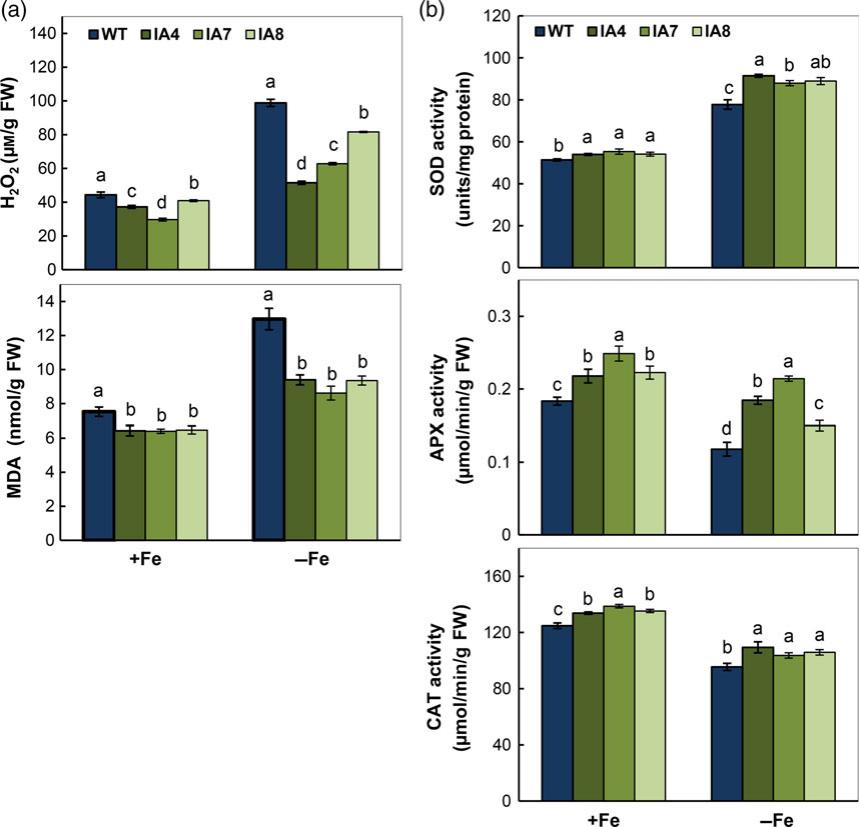
Antioxidant activity analysis in sweet potato plants under Fe‐sufficient (+Fe) and Fe‐deficient (−Fe) conditions. (a) H_2_O_2_ and malondialdehyde (MDA) content in the roots; (b) enzymatic activity of SOD, APX and CAT. IA,* IbVP1* transgenic line; WT, wild‐type plant. Values indicate means ± SD using three plants per line of three independent experiments. Different letters indicate significant differences (one‐way ANOVA,* P *<* *0.05).

As Fe is a cofactor of many antioxidant enzymes and generates reactive oxygen species (ROS) via Fenton reaction (Halliwell and Gutteridge, 1984), its deficiency leads to oxidative stress. Fe deficiency enhances H_2_O_2_ concentration and lipid peroxidation in roots of both IA transgenic and WT plants, particularly in the WT (Figure 9a). Compared with WT, the IA plants show less H_2_O_2_ concentration and lipid peroxidation. In parallel, the antioxidant activities of SOD, CAT and APX in IA lines are higher than in WT (Figure 9b), suggesting that overproduction of *IbVP1* leads to redox homoeostasis by up‐regulating ROS scavenging, especially under Fe deficiency. Nevertheless, all plants showed increased SOD activity and reduced APX and CAT activity, indicating differential response of ROS‐scavenging enzymes under Fe deficiency in sweet potato.

### Altered carbohydrate metabolism in *IbVP1* overexpressed sweet potato

**Figure 10:**
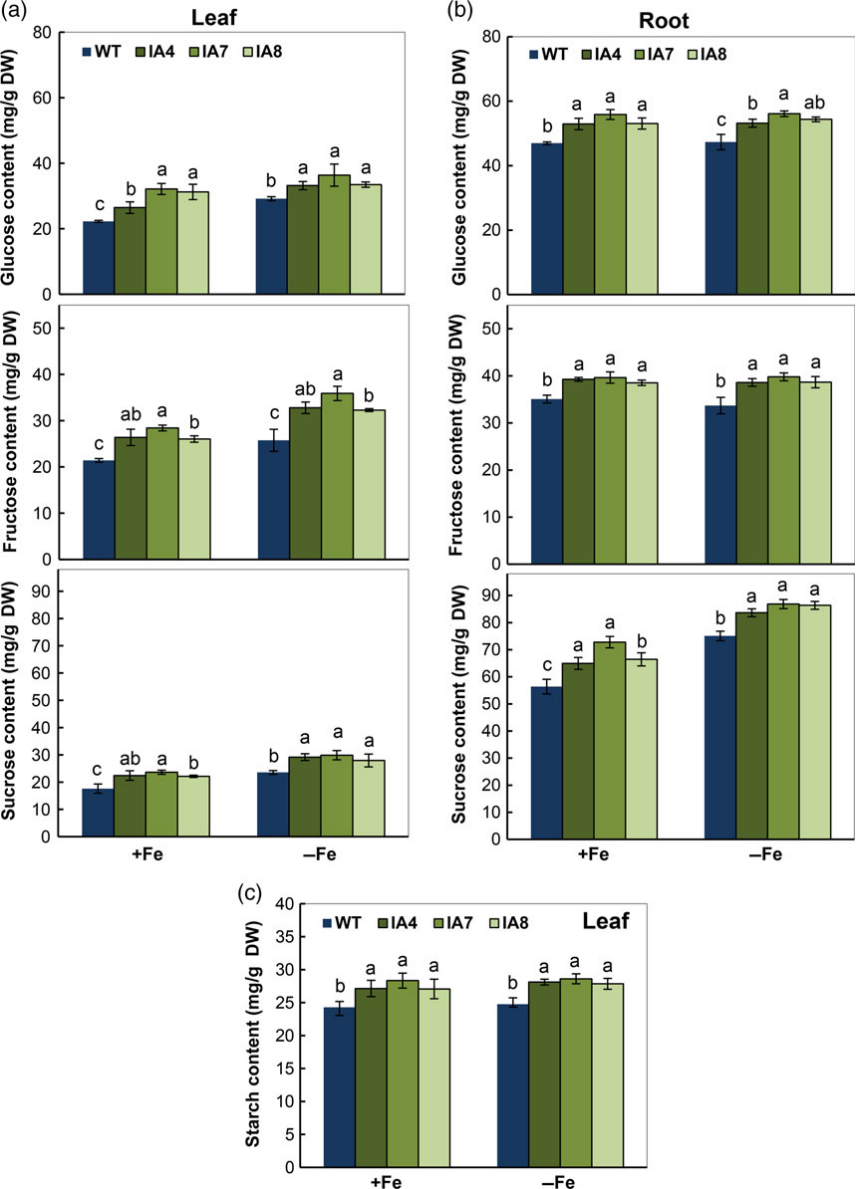
Changes in carbohydrate metabolism of sweet potato plants under Fe‐sufficient (+Fe) and Fe‐deficient (−Fe) conditions. (a, b) Glucose, fructose and sucrose levels in leaves (a) and roots (b); (c) starch content in leaves. IA,* IbVP1* transgenic line; WT, wild‐type plant. Values represent means ± SD using three plants per line of three independent experiments. Different letters indicate significant differences (one‐way ANOVA,* P *<* *0.05).

To enhance growth in the IA plants as stated previously (Figures 5, 6, 7), the glucose, fructose and sucrose levels in both leaves and roots, and starch content in leaves were measured. All the tissues were harvested at 12:00 pm. Irrespective of Fe levels, all transgenic plants contain relatively higher levels of all soluble sugars in leaves and roots compared with those in WT (Figure 10).

For example, under Fe‐deficient conditions, the fructose content was 32.3–35.9 mg/g DW in the leaves of transgenic plants, 25%–39% higher than in WT (25.8 mg/g DW). The glucose levels increased greatly under treatment for Fe deficiency: from 33.2 to 36.4 mg/g DW in the IA lines, 14%–25% higher than in WT. Higher sucrose levels in leaves and roots of transgenic plants were also observed. The sucrose levels increased significantly under Fe‐deficient conditions (Figure 10a,b). In leaves, relatively more starch was detected in the IA lines compared with WT (Figure 10c).

**Figure 11:**
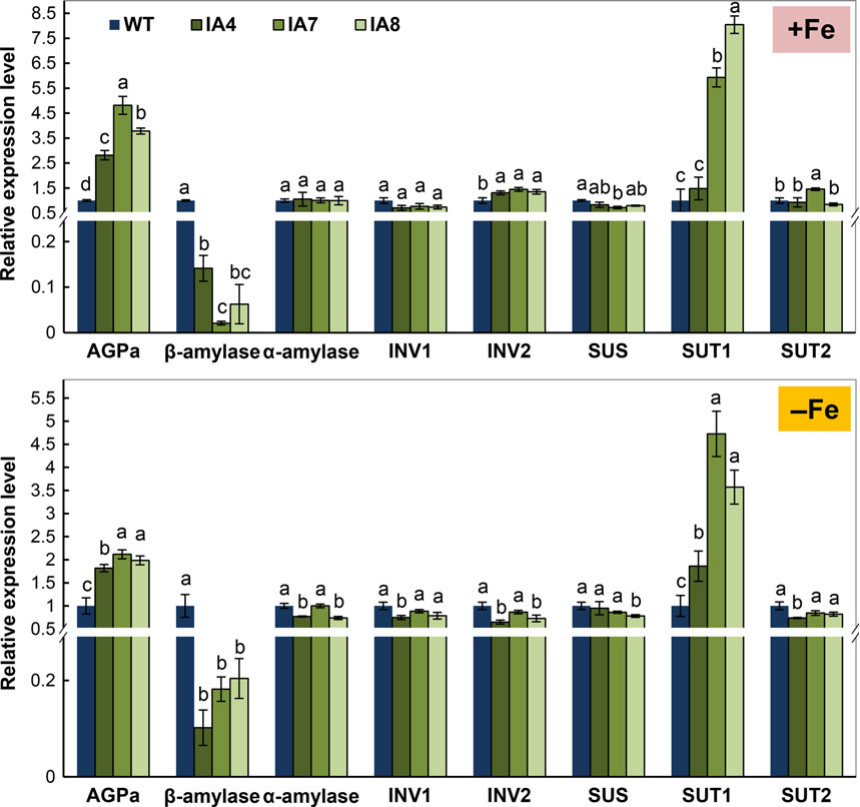
Expression patterns of sugar and starch metabolic genes in the roots of sweet potato plants under Fe‐sufficient (+Fe) and Fe‐deficient (−Fe) conditions. IA,* IbVP1* transgenic line; WT, wild‐type plant. AGPa, ADP‐glucose pyrophosphorylase; INV1, invertase 1; INV2, invertase 2; SUS, sucrose synthesis; SUT1, sucrose H^+^‐symporter; SUT2, sucrose transporter 2. qRT‐PCR data were normalized to those of the endogenous *Actin* gene. Error bars indicate the standard deviation between three technical replicates measured in fibrous roots collected from at least three different sweet potato plantlets and subsequently pooled for analysis. Different letters indicate significant differences (one‐way ANOVA,* P *<* *0.05).

Further expression analysis of genes related to sugar and starch metabolism in roots showed significant up‐regulation of *AGPase* and down‐regulation of β*‐amylase* in IA lines (Figure 11). Increased expression of *SUT1*, the sucrose transporter critical for cell‐to‐cell sucrose movement and sucrose phloem loading (Srivastava *et al*., 2008; Wang *et al*., 2015), was also demonstrated in the roots using qRT‐PCR (Figure 11). Other genes, such as α*‐amylase*,* INV1*,* INV2*,* SUS* and *SUT2*, were not affected in IA lines under either condition. These results indicate that carbon availability and allocation are affected by *IbVP1* overexpression in sweet potato.

## Discussion

Since the discovery of membrane‐bound H^+^‐PPases in plants (Karlsson, 1975), intensive studies of biological function in stress tolerance and plant development have been conducted. Under stressful conditions, up‐regulation of enzyme expression enables plant cells to use PPi as an energy source and maintain membrane integrity and intracellular transport via hydrolysis‐mediated ion transport (Gaxiola *et al*., 2016; Greenway and Gibbs, 2003; Stitt, 1998). Increased abiotic stress tolerance and enhanced growth performance in Pi or nitrogen deficiency were observed during H^+^‐PPase overexpression in Arabidopsis (Gaxiola *et al*., 2001), tomato(Dong *et al*., 2011; Park *et al*., 2005; Yang *et al*., 2007), tobacco (Khoudi *et al*., 2012), rice (Zhang *et al*., 2011; Zhao *et al*., 2006), maize (Li *et al*., 2008), lettuce (Paez‐Valencia *et al*., 2013) and cotton (Lv *et al*., 2009; Pasapula *et al*., 2011). Nevertheless, the role of H^+^‐PPase‐induced Fe uptake is unknown. In this study, we reported the role of *IbVP1* in improving Fe acquisition via increased rhizosphere acidification, carbohydrate metabolism and auxin regulation under Fe deficiency. Overexpression of *IbVP1* in sweet potato facilitates fortification of crop plants against Fe deficiency.

Sweet potato is mainly cultivated in semi‐arid and arid lands that lack bioavailable Fe, especially in alkaline calcareous soils. For example, in China, sweet potato is mainly grown in the mountainous and infertile sandy lands, which are prone to Fe deficiency (Zuo and Zhang, 2011). Iron is essential for plant growth, especially in the early and middle stages, which are key phases of aerial growth. A level of 33 mg Fe/kg DW in the young leaves of sweet potato represents the threshold for iron deficiency (O'Sullivan *et al*., 1997). Nevertheless, severe Fe deficiency in sweet potato is uncommon, possibly due to up‐regulated H^+^‐PPase expression, resulting in increased rhizosphere acidification and enhanced Fe acquisition, as shown in our study. Even under Fe deficiency, the Fe content in WT roots is approximately 1300 mg Fe/kg DW, which is 10 times higher than in WT leaves. Overexpression of *IbVP1* in sweet potato grown under Fe deficiency restored the Fe levels to normal values found in WT grown under Fe sufficiency. Therefore, IbVP1 is one of the key contributors to Fe acquisition in the species.

H^+^‐PPase has been considered as a yield‐enhancing factor (Gonzalez *et al*., 2010; Khadilkar *et al*., 2016). Increased biomass, especially root system, has been reported in plants overexpressing type I H^+^‐PPases, under deficiencies of phosphate (Gaxiola *et al*., 2012; Pei *et al*., 2012; Yang *et al*., 2007, 2014) and nitrogen (Lv *et al*., 2015; Paez‐Valencia *et al*., 2013). The type I H^+^‐PPase AVP1 triggers auxin efflux by up‐regulating the expression of Pinformed 1 auxin efflux facilitator and P‐adenosine triphosphatase, in Arabidopsis (Li *et al*., 2005) and *AUX1*,* PIN1a* and *PIN1b* in maize (Pei *et al*., 2012). In this study, the expression of *IbVP1* was also induced by auxin (IAA) treatment (Figure 2c,d). As auxin and its signalling pathways play a key function in plant response to Fe deficiency (Chen *et al*., 2010), application of exogenous auxin increased the expression of Fe acquisition genes, such as *FCR* in several plant species (Jin *et al*., 2011; Li *et al*., 2000; Zheng *et al*., 2003). The overexpression of *IbVP1* in sweet potato enlarged the root systems associated with increased IAA content in roots and significantly elevated the expression of auxin transport genes (i.e. *PIN1a*,* PIN1b* and *AUX1)* (Figure 4). As a result, auxin polar transport was increased in these IA lines. These results establish a relationship between Fe deficiency, *IbVP1* expression, auxin flux and root development in *IbVP1*‐overexpressing plants. Indeed, the expression of *IbVP1* was up‐regulated by IAA treatment (Figure 2) explaining the high yield of sweet potato grown in Fe‐deficient soils.

Enhanced rhizosphere acidification by increased PM‐H^+^‐ATPase activity is strongly associated with up‐regulated H^+^‐PPase activity, as demonstrated by the *IbVP1* overexpressing sweet potato under normal and Fe‐deficient conditions (Figures 3 and 7) consistent with other reports (Paez‐Valencia *et al*., 2013; Yang *et al*., 2007). Changes in the rhizosphere represent a central mechanism in plant mineral nutrition, contributing to nutrient solubility and proton‐motive force (Li *et al*., 2015; Palmgren, 2001; Palmgren, 1998; Santi and Schmidt, 2009; Zhu *et al*., 2009). The increase in root iron precipitation, Fe content and FCR activity in the IA lines demonstrated regulation of Fe uptake, which is associated with the up‐regulation of key genes such as *FRO2*,* IRT* and *FIT* involved in Fe acquisition (Figure 8).

Pyrophosphatases generate energy and release free Pi, which affects plant carbon metabolism (Gaxiola *et al*., 2012; Geigenberger *et al*., 1998). The altered partitioning of photoassimilate in tobacco and potato expressing an *Escherichia coli* cytosolic inorganic pyrophosphatase gene (Sonnewald, 1992) and the suppression of sucrose synthesis in the cotyledons of *avp* Arabidopsis mutant (Ferjani *et al*., 2011) support the significant role of PPases in sucrose metabolism. The concentrations of glucose, fructose and sucrose were significantly increased in leaves of IA transgenic lines grown in Fe‐sufficient or Fe‐deficient conditions (Figure 10). Accordingly, sugar transporter gene *SUT1*, which plays a critical role in sucrose phloem loading and transport as well as cellular sugar partitioning, was up‐regulated in the transgenic sweet potato (Figure 11). Further, the high expression of *IbVP1* in young storage root indicates an important function related to storage root development (Figure 2). These results further confirmed that up‐regulation of H^+^‐PPase promoted translocation of photosynthates from leaf to root, as hypothesized previously (Gaxiola *et al*., 2012; Hermans *et al*., 2006; Khadilkar *et al*., 2016; Paez‐Valencia *et al*., 2011). A recent study also demonstrated that increased sucrose accumulation was required for the regulation of auxin‐mediated Fe deficiency in plants (Lin *et al*., 2016). We hypothesize that the root phenotype of *IbVP1*‐overexpressing sweet potato is improved by increased auxin transport and enhanced sugar partitioning.

Fe deficiency in plants disrupts normal electron transfer in mitochondria and chloroplasts, resulting in overproduction of ROS and subsequent oxidative damage (Jelali *et al*., 2014; Ranieri *et al*., 2003; Tewari *et al*., 2005; Vigani *et al*., 2013). As an important cofactor, Fe is a constituent of antioxidant enzymes such as Fe‐SOD, catalase (CAT), ascorbate peroxidase (APX) and peroxidases (POD), and acts as a pro‐oxidant via the Fenton reaction (Ravet and Pilon, 2013). The activity of CAT or APX, both containing a heme group, was decreased under iron deficiency in plant species such as maize, sunflower, *Pyrus communis* and *Pisum sativum* (Donnini *et al*., 2011; Jelali *et al*., 2014; Ranieri *et al*., 2003; Sun *et al*., 2007). Therefore, enzyme activity is essential to maintain redox homeostasis in plants under stressful condition. Indeed, under Fe‐deficient conditions, decreased APX and CAT activities and increased SOD activity were detected in sweet potato, leading to accumulation of H_2_O_2_. In the IA lines, their activities were significantly higher than in WT, especially APX and CAT, showing improved ROS‐scavenging capacity (Figure 9), which is consistent with the previous reports (Jelali *et al*., 2014). The results confirmed that a high Fe availability facilitates detoxification of cellular enzyme systems.

In summary, our study suggests that *IbVP1* regulates Fe acquisition in sweet potato, and its overexpression promotes tolerance of transgenic plants to Fe deficiency. It induces plant growth via altered carbohydrate metabolism, improved auxin polar transport and increased rhizosphere acidification. This study also provides a rationale for induction of tolerance to iron deficiency in sweet potato grown in arid soils and provides a useful strategy for Fe acquisition in crops.

## Experimental procedures

### Plant materials, growth conditions and hydroponic treatments

The genome of sweet potato [*Ipomoea batatas* (L.) Lam.] Taizhong 6, a new farmer‐preferred cultivar released at our centre, was recently sequenced and released into public domain (http://public-genomes-ngs.molgen.mpg.de/SweetPotato, Yang *et al*., 2016). The cultivar was used in the experiment. Sweet potato plants were propagated by sprouts or vine cutting in field. Apical stems bearing 2–3 leaves were planted in plastic pots containing well‐mixed soil (soil : peat : perlite, 1 : 1 : 1) and grown in the greenhouse (16 h/8 h light/dark cycle, 25 °C day/night). *In vitro* shoot cultures were subcultured on SBM medium (MS salts including vitamins + 0.3 mg/L VB1 + 30 g/L sucrose, pH5.8) in plant growth chambers under a 16 h photoperiod provided by cool‐white fluorescent tubes (∼50 μmol/m^2^/s), at 25 °C and 50% relative humidity (RH).

Apical stems bearing two leaves and a petiole (two‐leaf plantlets) were harvested from sweet potato plants and incubated in brown flasks containing Fe‐EDTA nutrient solutions (+Fe, pH 6.5) for 2 weeks. The Fe‐EDTA nutrient solution had the following composition (in μm): Ca(NO_3_)_2_ (300), K_2_SO_4_ (50), MgSO_4_ (50), NaH_2_PO_4_ (30), H_3_BO_3_ (3), MnCl_2_ (0.5), ZnSO_4_ (0.4), CuSO_4_ (0.2), (NH_4_)_6_Mo_7_O_24_ (1) and Fe‐EDTA (100). The solution was refreshed every 2 days. After the development of the fibrous roots from the distal end of the petiole, the plantlets were transferred to nutrient solution that contained 0 μm Fe‐EDTA (−Fe, pH6.5) or hormone solutions for treatment. The plants were incubated with various concentrations (0, 50, 100, 200, 500 and 1000 μm) of 3‐indole acetic acid (IAA, Sigma, St Louis, MO, USA; 13750‐25G‐A) at 25 °C in the dark for 3 h. The plantlets were incubated in a 200 μm IAA solution at 25 °C in the dark for different durations (0–48 h).

### *IbVP1* cloning and sequence analyses

A sweet potato cDNA library was used to screen the H^+^‐PPase genes using *Arabidopsis thaliana* AVP1 cDNA probe (Genbank accession No. NM_101437). The full‐length cDNA was acquired by re‐sequencing the corresponding positive cDNA clones and submitted to NCBI under the Genbank accession No. JN688962.1. The deduced amino acid sequence of IbVP1 was aligned with H^+^‐PPases from other species. The molecular weight and isoelectric point (pI) of IbVP1 were predicted using the DNASTAR program (DNASTAR, Madison, WI). Sequence alignments were performed with ClustalW (http://www.ebi.ac.uk/clustalw, Chenna *et al*., 2003). The phylogenetic relationship of the sequences was analysed using the neighbour‐joining method with a bootstrap value of 500 replications under the Mega 6.0 program (Tamura *et al*., 2013). Transmembrane domains were predicted by the TMpred program (www.ch.embnet.org/software/TMPRED_form.html).

### Production of *IbVP1*‐overexpressed sweet potato plants

The PCR product of the full‐size *IbVP1* cDNA targeting *Kpn*I and *Sal*I restriction sites at the ends of the forward and reverse primers, respectively, was subcloned into the pMD‐18T vector ( TakaRa Dalian Co. Ltd., Dalian, China). After digestion with *Kpn*I and *Sal*I, the *IbVP1* cDNA segment was inserted into the pCAMBIA1301‐based plant expression vector to generate the binary vector pC1301‐IbVP1. The *Agrobacterium tumefaciens* strain LBA4404 harbouring the pC1301‐IbVP1 was used for sweet potato transformation. Shoot meristems from apical and axillary buds of sweet potato cultivar Taizhong 6 were used as material for embryogenic calli induction. Propagation, inoculation with *A. tumefaciens* strain and plant regeneration of embryonic calli were performed as described by Yang *et al*. (2011). Putative transgenic plants were monitored for rooting in the SBM medium supplemented with 10 mg/L hygromycin.

### Southern blot and qRT‐PCR analyses

Genomic DNA was isolated from plants cultured *in vitro* using the method described by Kim and Hamada (2005). Genomic DNA (20 μg) from wild type (WT) and transgenic lines was digested with corresponding restriction enzymes. After purification, the digested DNA samples were fractionated by 0.8% agarose gel and subsequently capillary transferred onto an Amersham Hybond N^+^ nylon membrane (GE Healthcare Life Sciences, Indianapolis, IN). The probes of *IbVP1* and hygromycin phosphor‐transferase gene (*HPT*) were labelled using a PCR DIG Probe Synthesis Kit (Roche Applied Science, Mannheim, Germany) with the primer pairs of *IbVP1* (VP1‐F and VP1‐R) and *HPT* (HPT‐F and HPT‐R) (Table S1). The hybridization with the DIG‐labelled oligonucleotide probes and the immunological detection were performed according to the manufacturer's instructions using the DIG‐High Prime DNA Labeling and Detection Starter Kit II (Roche Applied Science, Mannheim, Germany).

Extracted total RNA from the sweet potato leaves and roots using the RNAprep Pure Plant Kit (Tiangen, Beijing, China) was treated with DNase and reverse transcribed with M‐MLV Reverse Transcriptase RNaseH (Toyobo, Osaka, Japan). For the quantitative real‐time reverse transcription polymerase chain reaction (qRT‐PCR), specific primers of sweet potato genes (Table S1) were designed for analysing their expression levels using the SYBR Green PCR master mix (Toyobo, Osaka, Japan) in a Bio‐Rad CFX96 thermocycler. Amplification conditions were as follows: 95 °C for 1 min, followed by 40 cycles of 95 °C for 15 s and 60 °C for 30 s. To calibrate the expression level, the sweet potato *Actin* gene was chosen as the internal control.

Semi‐quantitative RT‐PCR was performed under the following amplification conditions for the *IbVP1* fragment (using similar primers as in the qRT‐PCR): initial denaturation at 94 °C for 5 min, followed by 30 cycles of 94 °C for 30 s, 60 °C for 30 s, 72 °C for 30 s and then a final extension at 72 °C for 10 min. Amplification conditions for the actin fragment were as follows: initial denaturation at 94 °C for 4 min, followed by 26 cycles of 94 °C for 30 s, 60 °C for 30 s, 72 °C for 30 s and followed by a final extension at 72 °C for 10 min. The PCR products were separated by 1.2% (w/v) agarose gel electrophoresis.

### Vacuole isolation and vacuolar H^+^‐PPase activity measurement

For measurement of vacuolar H^+^‐PPase activity, sweet potato root tonoplast vesicles were isolated by sucrose density gradient ultracentrifugation according to the method of De Michelis and Spanswick (1986). Root tip segments (~20 g) were harvested and rinsed in cold distilled water, and then homogenized in 80 mL of ice‐cold buffer containing 50 mm HEPES/Tris (pH 7.8), 10% glycerol, 2 mm EGTA, 0.25 m sucrose and 0.5% BSA (w/v). After filtration, precentrifugation of the homogenate was performed at 4200 g for 10 min to eliminate cell debris. The supernatant was then centrifuged at 50228 ***g*** for 2 h in a BECKMAN XL‐70 (Beckman, Palo Alto, CA, USA). After gently resuspension, the microsomal suspension was layered directly onto discontinuous sucrose gradients and centrifuged at 100 000 ***g*** for 2 h; the collected pellets were resuspended. After centrifugation at 146 000 ***g*** for 1 h, the vesicle pellets were collected and resuspended in storage buffer containing 5 mm HEPES/Tris (pH 7.4), 2 mm DTT and 2 mm MgSO_4_. V‐H^+^‐PPase activities were determined by the release of Pi from PPi after incubation for 30 min at 25 °C. Each of the activity measurements used approximately 20 μg of protein. Pi determination was based on the method of Lin and Morales (1977). V‐H^+^‐PPase hydrolytic activity was calculated as the difference in values measured in the presence and absence of 50 mm KCl (K^+^‐stimulated PPase activity).

### Plasma membrane vesicle isolation and PM‐ATPase assay

Plasma membrane vesicles were isolated from sweet potato roots using the phase partitioning method reported by Klobus and Buczek (1995). Sweet potato roots (10 g) were used for the isolation. PM‐H^+^‐ATPase hydrolytic activity was measured as described by Janicka‐Russak *et al*. (2008). Fifty‐microlitre membrane vesicle preparations were added to 0.5 mL reaction solution containing ATP in a tube and were incubated at 37 °C for 20 min. Absorption at 660 nm (A660) was measured with Beckman Coulter DU730 (Fullerton, CA, USA) spectrophotometer. The net PM‐ATPase A660 was calculated by subtracting the A660 with Na_3_VO_3_ from A660 without Na_3_VO_3_. The enzyme activity was calculated according to the protein and inorganic phosphorus content obtained and the reaction time (20 min).

### Root ferric reductase activity, acidification capacity and Perls’ Prussian blue staining

Ferric reductase activity was determined using the method of Grusak and Pezeshgi (1996). Briefly, 0.1 g of the whole excised root was placed in a tube filled with 10 mL of assay solution, which consisted of 0.5 mm CaSO_4_, 0.1 mm BPDS, 0.1 mm MES and 100 μm Fe‐EDTA at pH 5.5. During storage in a dark room at 25 °C for 2 h, the tubes were periodically hand‐swirled at 20‐min intervals. The absorbance of the assay solutions was measured at 535 nm, and the concentration of Fe (II) [BPDS]_3_ was quantified using a standard curve.

Acidification capacity was detected using the method of Yi and Guerinot (1996). Briefly, one‐week‐old subculture plantlets grown on agar plastic jars were transferred to Fe‐deficient medium for 3 d, and the plantlets were transferred to a 1% agar plate containing 0.006% bromocresol purple and 0.2 mm CaSO_4_ (pH 6.5) for 4 h.

To localize Fe^3+^, 2‐week‐old plantlets grown on Fe‐sufficient medium were vacuum‐infiltrated with fresh Perls’ Prussian blue staining solution (equal volumes of 4% (w/v) potassium ferrocyanide and 4% (v/v) HCl) for 30 min. After rinsing with water, plantlets were observed and photographed.

### Chlorophyll content measurement

The chlorophyll content of young leaves was detected with the Minolta SPAD‐502 leaf chlorophyll meter using the SPAD reading. To compensate for leaf variables, the transmittance at 940 nm served as a reference, as chlorophyll concentration influences the absorption at 650 nm (Azia and Stewart, 2001). For spectrophotometric determination of chlorophyll content, the leaf samples (200 mg) collected from sweet potato plants were homogenized with 95% ethanol (v/v) and kept in dark for 2 days. After filtering through a filter paper, the homogenate was used for the total chlorophyll content determination as described by Arnon (1949).

### Sugar and starch content analysis

Materials harvested at 12:00 pm from WT and transgenic plants were ground and baked at 80 °C for two days until a constant dry weight (DW). The dried sample 30 mg was dissolved in 0.7 mL of 80% ethanol, thoroughly vortexed and incubated at 70 °C for 2 h. Aliquots of 0.7 mL HPLC‐grade water and 0.7 mL chloroform were added to the sample. After shaking several times, the mixtures were centrifuged at 12 000 ***g*** for 10 min. The pellet was analysed for starch content while the supernatant was composed of soluble sugar. The starch pellet was washed three times with 80% ethanol, and the total starch content was analysed using total starch kit (Megazyme International Ireland Limited, Wicklow, Ireland). The aqueous supernatant of 0.7 mL was transferred into 1.5‐mL tube and mixed with 0.7 mL chloroform. After centrifugation at 12 000 ***g*** for 10 min, 0.5 mL of the supernatant was transferred to a glass tube for HPLC analysis of sugars. The sugar separation was carried out using the Agilent HPLC column (ZORBAX Carbohydrate column; 4.6 × 150 mm, 5 μm) with a differential refraction detector. The mobile phase consisted of 75% acetonitrile with a flow rate of 0.8 mL/min, and the column was kept at 35 °C. The sugar types were characterized according to the retention time of the standard references. The concentration in samples was calculated from the external standard curve.

### IAA measurement

3‐indole acetic acid was extracted as described by Pan *et al*. (2010) with modifications. Using a mortar and pestle, 0.5 g new fresh tissue from sweet potato plants was ground in fine powder in liquid nitrogen. After incubation with 5 mL extraction solvent (β‐propanol:H_2_O:concentrated HCl, 2 : 1 : 0.002, v/v/v) for 30 min at 4 °C under continuous shaking in the dark, 5 mL dichloromethane was added to each sample and vortexed for 30 min in a cold room at 4 °C. The samples were transferred to a refrigerated microcentrifuge at 4 °C and centrifuged at 13 000 ***g*** for 5 min. About 4.5 mL of the solvent from the lower phase was transferred into a screwcap vial using a Pasteur pipette, and the solvent mixture was concentrated (not completely dry) using N_2_ gas. The samples were re‐dissolved in 0.1 mL methanol. The IAA ELISA kit was used for IAA content measurement (IAA ELISA kit, Sigma, St Louis, MO, USA). The samples were measured four times, and the standard error was calculated.

### Metal content assay

Sweet potato seedlings grown in hydroponics were washed as described (Gong *et al*., 2003) before sampling root and leaf tissues. The control plant tissues and plant tissues treated for Fe deficiency were digested in concentrated nitric acid for 5–7 days at room temperature, and the samples were boiled and completely digested. After dilution with Millipore‐filtered deionized water, samples were briefly centrifuged. The diluted samples were measured using ICP‐Mass spectrometer (ELAN DRC‐e, Perkin Elmer, Toronto, ON, Canada).

### Measurement of lipid peroxidation and H_2_O_2_ content

Lipid peroxidation in the leaf tissues (200 mg) from the sweet potato plants was assayed for malondialdehyde (MDA) as essentially described by Dhindsa and Matowe (1981). The H_2_O_2_ content was assessed using 1 g leaf tissues according to the method of Sairam and Srivastava (2002). All analyses were carried out in triplicate.

### ROS‐scavenging enzyme activity assays

Superoxide dismutase activity was assessed using the method of Beauchamp and Fridovich (1971), by measuring the photochemical inhibition of NBT at 560 nm. CAT activity was measured according to Xu *et al*. (2013) by monitoring the consumption of H_2_O_2_ at 240 nm for 4 min. The APX activity was determined according to Xu *et al*. (2014). Ascorbate oxidation was measured spectrophotometrically based on the decrease in absorption at 290 nm, using the absorption coefficient of 2.8/mm/cm. All measurements were conducted in triplicate.

### Statistical analysis

All data were represented as mean ± SD from at least three independent experiments with three replicates each. Significant differences between treatments were analysed with one‐way analysis of variance (ANOVA) using the program SigmaPlot 10.0 (Systat Software, San Jose, CA). A value of *P* < 0.05 was considered statistically significant difference.

## Supporting information


**Figure S1** Southern blotting analysis of the *IbVP1* gene in sweet potato genome.
**Figure S2** Semi‐quantitative RT‐PCR analysis of *IbVP1* expression in sweet potato and in response to Fe deficiency and auxin treatment.
**Figure S3** Generation and molecular analysis of *IbVP1*‐overexpressed transgenic sweet potato plants.
**Table S1** Primers used for probe amplification and qRT‐PCR analysis.
