# Supplementary material for: H+‐pyrophosphatase IbVP1 promotes efficient iron use in sweet potato [Ipomoea batatas (L.) Lam.]
Source: Plant Biotechnol J. 2017 Feb 10;15(6):698–712. doi: 10.1111/pbi.12667 (PMC5425394; doi:10.1111/pbi.12667)
Supplement: Supplementary file 1 — Figure S1 Southern blotting analysis of the IbVP1 gene in sweet potato genome. Figure S2 Semi‐quantitative RT‐PCR analysis of IbVP1 expression in sweet potato and in response to Fe deficiency and auxin treatment. Figure S3 Generation and molecular analysis of IbVP1‐overexpressed transgenic sweet potato plants. Table S1 Primers used for probe amplification and qRT‐PCR analysis. [file PBI-15-698-s001.docx]

**Supporting Information**

**Supplementary Table**

**Table S1.** Primers used for probe amplification and qRT-PCR analysis.

| **Gene** | **Primer** | **Sequence (5'- 3')** |
| --- | --- | --- |
| ***IbVP1*** | VP1-F | GGGCTTCAGAACATGCGAGAACTC |
|  | VP1-R | CGTACAAAATGACAAATGTACT |
| ***HPT*** | HPT-F | TTCTACACAGCCATCGGTCC |
|  | HPT-R | CCCATGTGTATCACTGGCAA |
| ***Actin*** | Actin-F | TTCCGATCTCTCTCGCACTC |
|  | Actin-R | CCTCTTCTGCCATCTTCTGC |
| ***AGPa*** | AGPa-F | TCGACGGTGATGTTAGCAAG |
|  | AGPa-R | AACAGCCTTTGGAGAAACGA |
| ***β-amlyase*** | β-amlyase -F | AGACTGGAAGGAGGCTGTGA |
|  | β-amlyase -R | TGTTGGCTTCTTCGAGGACT |
| ***α-amlyase*** | α-amlyase -F | CTGCATTTTTGTTCCTGCAA |
|  | α-amlyase -R | TTCGATGCGTCCAAGTCATA |
| ***INV1*** | INV1-F | TGATGAATGGGGAACCTGAT |
|  | INV1-R | GTCCACTGGAGCGACTCTTC |
| ***INV2*** | INV2-F | TGATGAATGGGGAACCTGAT |
|  | INV2-R | GTCCACTGGAGCGACTCTTC |
| ***SUS*** | SUS-F | CCTGCCAATGGTAACTTCGT |
|  | SUS-R | CGTCTTGCCCTTGTATTGGT |
| ***SUT1*** | SUTI-F | TGGTCTCTCCCTTGGTGTTC |
|  | SUTI-R | GATTGAGGTTTGGGAAGCAA |
| ***SUT2*** | SUT2-F | GAGCGAAGAGGATGAGAACG |
|  | SUT2-R | CCAGGGTAGTCACGGACAGT |
| ***AHA2*** | AHA2-F | AGACGAGCATCAGCTGGAAT |
|  | AHA2-R | GAAGAAAACAATGCCGGAAA |
| ***FRO2*** | FRO2-F | CTTTGCTTGCCACACTTGAA |
|  | FRO2-R | GGGAAAAGGGATCACCAAAT |
| ***IRT*** | IRT-F | TTATGGCGCTGTTTTTCTC |
|  | IRT-R | GCTTGGGATTCTTGTGCATT |
| ***FIT*** | FIT-F | GGTTTGTGAACGGAAGAGGA |
|  | FIT-R | TTGCTATTTCCGCCTTGAGT |
| ***PIN1a*** | PIN1a -F | CACATGTTTGTTTGGAGCTC |
|  | PIN1a -R | TCATTTCTGTATCTCTGTTC |
| ***PIN1b*** | PIN1b -F | GGCTTTCTGGGAACTGTCAA |
|  | PIN1b -R | TAGGAGTTGGGGTTCCTTGA |
| ***AUX1*** | AUX1 -F | TTTCTCAAATGGGGATGCTC |
|  | AUX1 -R | AACAAGCCATCCAACACCTC |

**Supplementary Figures**

**
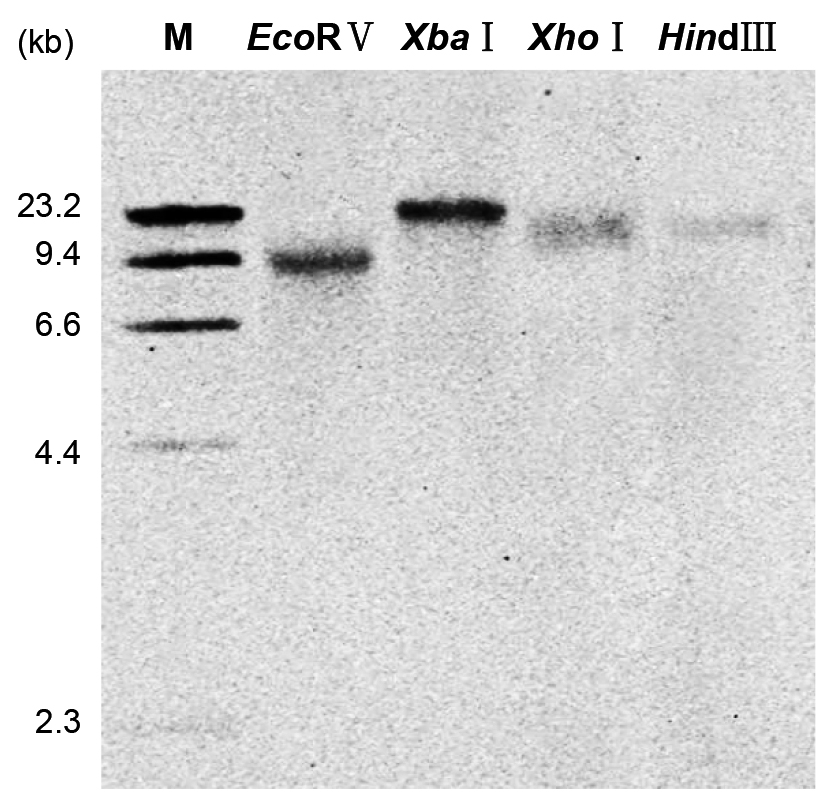
**

**Figure S1.** Southern blotting analysis of the *IbVP1*gene in sweet potato genome. Genomic DNA was digested by *Eco*RV, *Xba*I, *Xho*I and *Hin*dIII, respectively, and the gel blot was hybridized with a DIG-labeled probe for *IbVP1*.


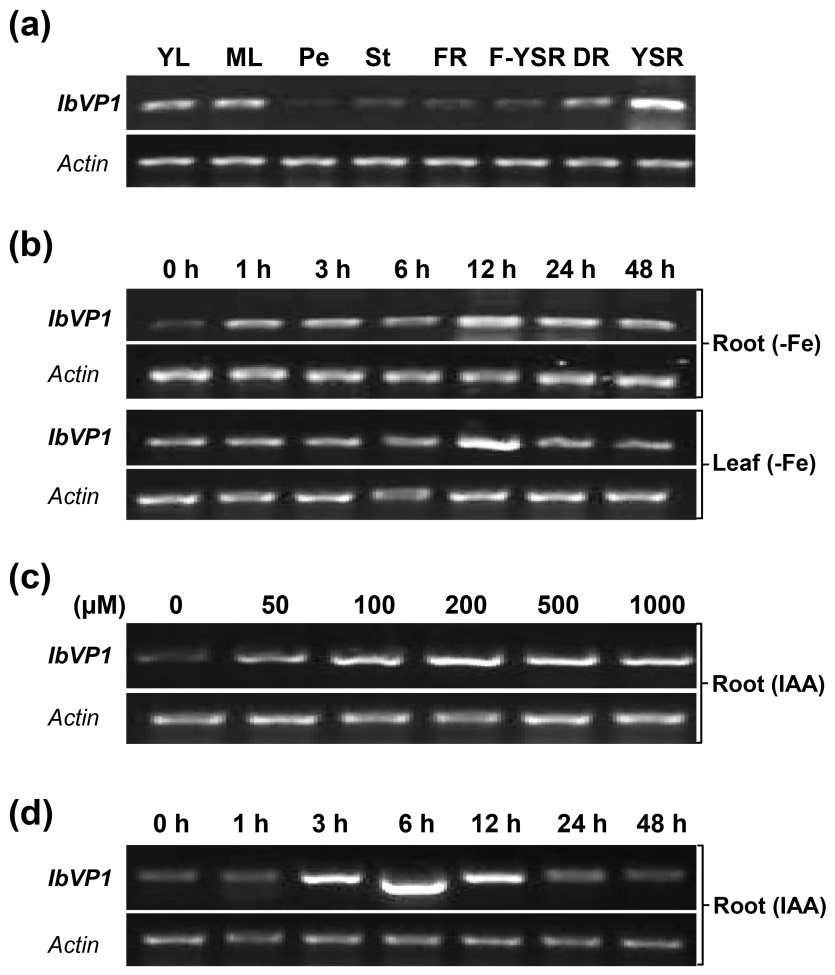


**Figure S2.** Semi-quantitative RT-PCR analysis of *IbVP1* expression in sweet potato and in response to Fe deficiency (-Fe) and auxin (IAA) treatment. (a) Expression pattern of *IbVP1* in tissues of plants at 50 d after planting; YL, young leaf; ML, mature leaf; Pe, petiole; St, stem; FR, fibrous root (diameter < 0.2 cm); F-YSR, fibrous root from young storage root; DR, developing root (0.2 cm < diameter < 0.5 cm); YSR, young storage root (diameter 0.5–1.0 cm). (b) Time course response of *IbVP1* expression in sweet potato under –Fe condition in fibrous roots and leaves at various time periods (0–48 h); (c) Transcriptional variation in *IbVP1* in response to treatment with various concentrations of exogenous IAA for 3 h in sweet potato roots; (d) *IbVP1* expression in response to IAA (200 µM) for various time periods (0–48 h) in sweet potato roots. The sweet potato *Actin* gene was used as a control.


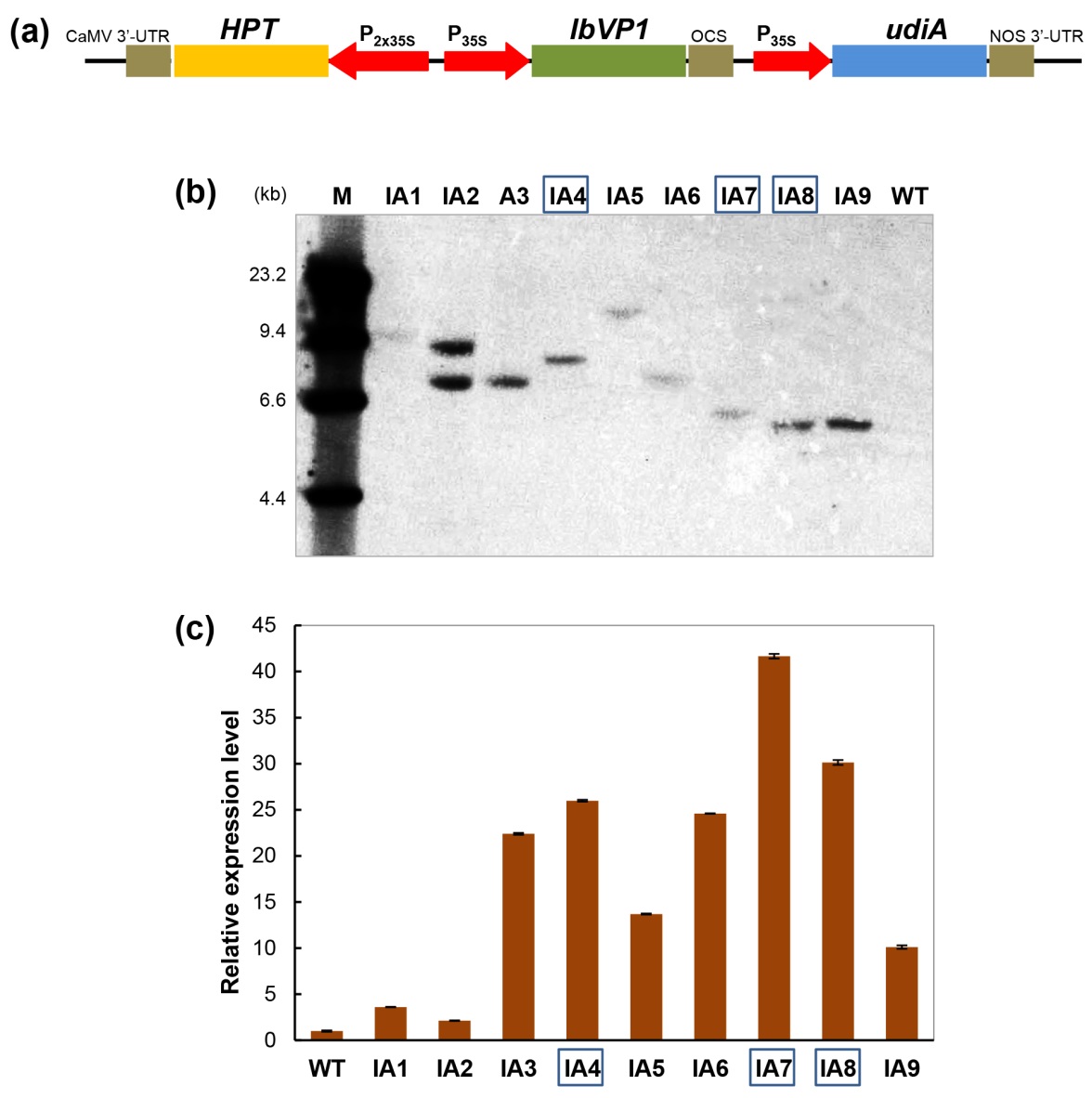


**Figure S3.** Generation and molecular analysis of *IbVP1*-overexpressed transgenic sweet potato plants. (a) Schematic map of T-DNA region in the pC1301-IbVP1 vector used for sweet potato transformation. P_35S_: CaMV35S promoter; OCS, octopine synthase gene terminator; *HPT*, hygromycin phosphotransferase gene; *udiA*, β-glucuronidase gene. (b) Southern blot of transgenic plant lines (IA) and wild type (WT). Sweet potato genomic DNA was digested with restriction enzyme *Eco*RI that cuts once within the T-DNA region and was hybridized with DIG-labelled *HPT* probe. (c) qRT-PCR analyses of *IbVP1* expression in different transgenic lines. M, molecular marker; IA1-IA9, independent *IbVP1-*expressing transgenic lines; WT, wild type.
